# Toward a Recognition System for Mexican Sign Language: Arm Movement Detection

**DOI:** 10.3390/s25123636

**Published:** 2025-06-10

**Authors:** Gabriela Hilario-Acuapan, Keny Ordaz-Hernández, Mario Castelán, Ismael Lopez-Juarez

**Affiliations:** Robotics and Advanced Manufacturing Department, Centre for Research and Advanced Studies (CINVESTAV), Ramos Arizpe 25900, Mexico; gabriela.hilario@cinvestav.mx (G.H.-A.); mario.castelan@cinvestav.mx (M.C.)

**Keywords:** Mexican Sign Language, dynamic signs, pattern analysis, pose-based approach, computer vision, machine learning, CNN, YOLOv8, arm movement

## Abstract

This paper describes ongoing work surrounding the creation of a recognition system for Mexican Sign Language (LSM). We propose a general sign decomposition that is divided into three parts, i.e., hand configuration (HC), arm movement (AM), and non-hand gestures (NHGs). This paper focuses on the AM features and reports the approach created to analyze visual patterns in arm joint movements (wrists, shoulders, and elbows). For this research, a proprietary dataset—one that does not limit the recognition of arm movements—was developed, with active participation from the deaf community and LSM experts. We analyzed two case studies involving three sign subsets. For each sign, the pose was extracted to generate shapes of the joint paths during the arm movements and fed to a CNN classifier. YOLOv8 was used for pose estimation and visual pattern classification purposes. The proposed approach, based on pose estimation, shows promising results for constructing CNN models to classify a wide range of signs.

## 1. Introduction

Deafness or hearing loss is the partial or total loss of the ability to hear sounds in one or both ears. The World Health Organization’s most recent World Hearing Report [1] estimates that more than 1.5 billion people have some degree of hearing loss. Approximately 430 million of them have moderate or greater hearing loss in their better ear; this number is expected to increase to 700 million people by 2050.

According to the Ministry of Health [2], approximately 2.3 million people in Mexico have hearing disabilities. This vulnerable group faces significant levels of discrimination and limited employment opportunities. Additionally, this health condition restricts access to education, healthcare, and legal services, further exacerbating social inequalities and limiting opportunities for integration. One of the primary challenges faced by the deaf community is communication with hearing individuals, as linguistic differences hinder social and workplace interactions. While technology has proven useful in reducing some of these barriers, deaf individuals often rely on the same technological tools as the hearing population, such as email and text messaging applications. However, these tools are not always effective, as not all deaf individuals are proficient in written Spanish.

In the Americas, the most widely studied sign languages are American Sign Language (ASL) and Brazilian Sign Language (LIBRAS), which have facilitated research and technological advancements aimed at improving communication with the deaf community. An example of one innovation is SLAIT [3], a startup that emerged from a research project at Aachen University of Applied Sciences in Germany. During this research, an ASL recognition engine was developed using MediaPipe and recurrent neural networks (RNNs). Similarly, Ref. [4] announced an innovative project in Brazil that uses computer vision and artificial intelligence to translate LIBRAS into text and speech in real time. Although this technology is still undergoing internal testing, the developers claim that after four years of work, the system has reached a significant level of maturity. This technology was developed by Lenovo researchers in collaboration with the Center for Advanced Studies and Systems in Recife (CESAR), which has already patented part of this technology [5]. The system is capable of recognizing the positions of arm joints, fingers, and specific points on the face, similar to SLAIT. From this data, it processes facial movements and gestures, enabling the identification of sentence flow and the conversion of sign language into text. CESAR and Lenovo believe that their system has the potential to become a universally applicable tool.

Compared to speech recognition and text translation systems, applications dedicated to sign language (SL) translation remain scarce. This is partly due to the relatively new nature of the field and the inherent complexity of sign language recognition (SLR), which involves visual, spatial, and gestural elements. Recognizing sign language presents a significant challenge, primarily due to limited research and funding. This highlights the importance of promoting research into the development of digital solutions that enhance the quality of life for the deaf community (c.f. [6]). However, researchers agree that the key factor for developing successful machine learning models is data (c.f. [7]). In this regard, for SLs like LSM, existing databases are often inadequate in terms of both size and quality, hindering the advancement of these technologies. Also, sensing technology has a fundamental role in the reliability of incoming data. This is the main reason why SLR is broadly divided into two branches, i.e., contact sensing and contactless sensing.

Sign data acquisition with contact relies on gloves [8], armbands [9], wearable inertial sensors [10,11], or electromyographic (EMG) signals [12]. In contrast, contactless sign data acquisition is mainly divided into two types, depending on the kind of hardware, that is, simple hardware (color or infrared cameras) or specialized hardware (e.g., depth sensors, optical 3D sensors [13], commercial WiFi devices [14], and ultrasonic devices [15]).

**Figure 1:**
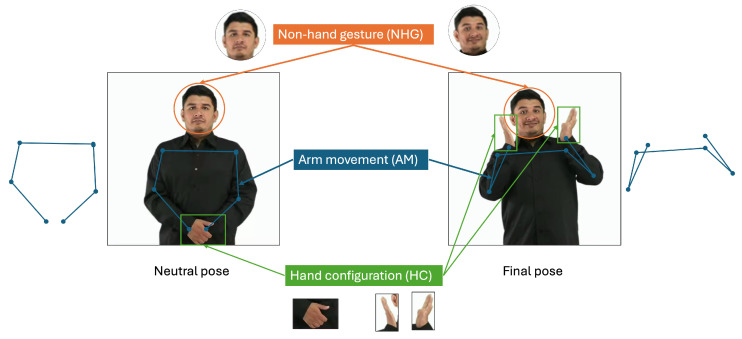
Sign features: hand configuration (HC), arm movement (AM), and non-hand gesture (NHG). “Surprise!” sign images were taken from screenshots of the corresponding YouTube video of the GDLSM [38]; see Appendix A.

This classification is similar to the one presented by [16] (Figure 1), except that their sign data acquisition approaches are divided into sensor-based approaches and vision-based approaches. We present several examples of sign language research and related work, along with various approaches to sign data acquisition, as detailed in Table 1.

In Table 1, we include information regarding the features of signs that are included in the sign data acquisition for each reported work. Instead of using the separation employed by [17] (facial, body, and hand features), we propose our own decomposition into hand configurations (HCs), arm movements (AMs), and non-hand gestures (NHGs); see Figure 1. This is a fundamental concept of our research, so this decomposition is discussed in more detail in Section 1.1.2. The separation of facial, body, and hand features is a concept commonly seen in pose estimators (such as MediaPipe [18]), which are also common in SL research, as presented in Table 1. It is also possible to observe that most SL research is focused on the HC features.

We will now present the scientific context of LSM research. First, we present the known datasets and then present studies about LSM recognition and analysis.

LSM is composed of two parts, i.e., dactylology (fingerspelling) and ideograms ([19], p. 12). Dactylology is a small subset of LSM and basically consists of letters of the alphabet, where most parts are static signs. A few signs for numbers are also static. Due to the small, nevertheless important, role of dactylology, we are interested in LSM ideogram datasets. To the best of our knowledge, there are three publicly available ideogram-focused datasets. Two of them are visual, i.e., (i) the MX-ITESO-100 preview [20], which contains video clips of 11 signs from 3 signers (out of 100 signs, although not all are currently available), and (ii) the Mexican Sign Language dataset [21,22], which includes image sequences of 249 signs from 11 signers. The third dataset, consisting of keypoints, is provided by [23]; this dataset contains 3000 samples of 30 signs from 4 signers (8 letters, 20 words, and 2 phrases). This was constructed by processing the RGBD data into keypoints by means of the MediaPipe [18] tool, but the unprocessed visual data is not provided. A comparison of these datasets, along with LSM glossaries, is provided in Table 2 and Table 3.

**Table 1:** Sign language research and related work.

Ref.	SL	Sign Group *	Sign Type	Sign Features ^†^	Sensor/Tool
Yao et al. (2025) [24]	ASL	L, N, P	Static	HC	Hydrogel strain sensor
Chiradeja et al. (2025) [8]	-	S	Dynamic	HC	Gloves
Rodríguez-Tapia et al. (2019) [10]	ASL	W	Dynamic	HC	Myoelectric bracelets
Filipowska et al. (2024) [12]	PJM	W	Dynamic	HC	EMG
Umut and Kumdereli (2024) [9]	TSL	W	Dynamic	HC, AM	Myo armbands (IMU + sEMG)
Gu et al. (2024) [11]	ASL	W, S	Dynamic	HC, AM	IMUs
Wei et al. (2025) [25]	-	W	Dynamic	HC	Gloves
Wang et al. (2025) [26]	ASL	L	Both	HC	Triboelectric sensor
Urrea et al. (2023) [27]	ASL	L, W	Static	HC	Camera/MediaPipe
Al-Saidi et al. (2024) [16]	ArSL	L	Static	HC	Camera/MediaPipe
Niu (2025) [28]	ASL	L	Static	HC	Camera
Hao et al. (2020) [14]	-	W	Dynamic	HC	WiFi
Galván-Ruiz et al. (2023) [13]	LSE	W	Dynamic	HC	Leap motion
Wang et al. (2023) [15]	CSL	W, P	Dynamic	HC	Ultrasonic
Raihan et al. (2024) [29]	BdSL	L, N, W, P	Dynamic	HC	Kinect
Woods and Rana (2023) [30]	ASL	W	Dynamic	AM, NHG	Camera/OpenPose
Eunice et al. (2023) [31]	ASL	W	Dynamic	HC, AM, NHG	Camera/Sign2Pose, YOLOv3
Gao et al. (2024) [17]	ASL, TSL	W	Dynamic	HC, AM, NHG	Camera, Kinect
Kim and Baek (2023) [32]	DGS, KSL	W, S	Dynamic	HC, AM, NHG	Camera/AlphaPose
Boháček and Hrúz (2022) [33]	ASL, LSA	W	Dynamic	HC, AM, NHG	Camera/Vision API (Apple)
Cihan Camgöz et al. (2020) [34]	DGS	S	Dynamic	HC, AM, NHG	Camera
Miah et al. (2024) [35]	ASL, PSL, LSM	L, W, P	Dynamic	HC, AM, NHG	Camera/MediaPipe, OpenPose
Gil-Martín et al. (2023) [36]	LSE	L, N, W	Both	HC, AM, NHG	Virtual ^‡^ camera/MediaPipe
Villa-Monedero et al. (2023) [37]	LSE	L, N, W	Both	HC, AM, NHG	Virtual ^‡^ camera/MediaPipe
Current study	LSM	W, P	Dynamic	AM	Camera/YOLOv8

* L: alphabet letter; N: number; W: word; P: phrase; S: sentence; ^†^ HC: hand configuration; AM: arm movement; NHG: non-hand gesture. ^‡^ Virtual camera since the dataset was created using synthetic avatars. SL names are provided in the Abbreviations section. Top part: Sign data acquisition with contact sensing. Bottom part: Contactless sign data acquisition.

**Table 2:** LSM datasets and glossaries.

Ref.	Type	Sign Group *	Sign Signal	Samples
DIELSEME 1 (2004) [39]	Glossary ^†^	535 W	Visual	1 video per sign
DIELSEME 2 (2009) [40]	Glossary ^†^	285 W	Visual	1 video per sign
GDLSM (2024) [38]	Glossary	27 L, 49 N, 667 W, 4 P	Visual	1 video per sign ^‡^
MX-ITESO-100 (2023) [20]	Dataset	96 W, 4 P	Visual	50 videos per sign
Mexican Sign Language dataset (2024) [22]	Dataset	243 W, 6 P	Visual	11 image sequences per sign
Mexican Sign Language Recognition (2022) [23]	Dataset	8 L, 21 W, 1 P	Keypoints	100 samples per sign

* L: alphabet letter; N: number; W: word; P: phrase. ^†^ According to [41], DIELSEME 1 and 2 are actually glossaries and not dictionaries. The three LSM glossaries contain only one sample per sign, whereas the datasets include multiple samples per sign. ^‡^ Their site reports 719 videos, but only 715 were found; also, the 32 videos in the “*Estados y capitales*” thematic category include 2 signs per video.

**Table 3:** LSM datasets and glossaries: sign and signal properties.

Ref.	Sign Features	Signal Properties	File Format	Comments
DIELSEME 1 (2004) [39]	HC, AM *, NHG	320 × 234 @ 12 fps	SWF videos	
DIELSEME 2 (2009) [40]	HC, AM, NHG	720 × 405 @ 30 fps	FLV videos	
GDLSM (2024) [38]	HC, AM, NHG	1920 × 1080 @ 60 fps	videos	Hosted on a streaming platform; c.f. Appendix A
MX-ITESO-100 (2023) [20]	HC, AM, NHG	512 × 512 @ 30 fps	MP4 videos	Preview only ^‡^
Mexican Sign Language dataset (2024) [22]	HC, AM *	640 × 480	JPEG images	Blurred faces
Mexican Sign Language Recognition (2022) [23]	HC, AM, NHG	20 × 201 array	CSV files	One row per frame, 67 (x,y,z) keypoints

* In those cases, the background and clothing are black, so the segmentation of skin (hand and face) is easier, but tracking joints for AM is more difficult. ^‡^ Only 11 signs (words) are available in the public preview. Also, the 50 samples of each sign were performed by a single subject.

Regarding LSM studies, most of the SLR research on LSM mainly focuses on classifying static letters and numbers using classical machine learning techniques and convolutional neural networks (CNNs) [42,43,44,45,46,47,48,49]. Using the classification provided by [16], there are four classes of signs: (i) continuous signs, (ii) isolated signs, (iii) letter signs, and (iv) number signs. In LSM, most of the signs in the last three categories are static signs. But signing in LSM is generally highly dynamic and continuous, since most signs are ideograms, as mentioned before.

In terms of dynamic sign recognition, early studies focused on classifying letters and numbers with motion. For example, Ref. [50] used the CamShift algorithm to track the hand trajectory, generating a bitmap that captures the pixels of the hand path; these bitmaps were then classified using a CNN. Another approach, presented in [51], involved obtaining the coordinates (*x*,*y*) of 22 keypoints of the hand using an Intel RealSense sensor, which were used as training data for a multilayer perceptron (MLP) neural network. Finally, in [52], 3D body cue points obtained with MediaPipe were used to train two recurrent neural networks (RNNs), i.e., LSTM and GRU.

In more recent research, in addition to letters and numbers, some simple words and phrases were included. Studies such as Refs. [53,54,55] used MLP-type neural networks, while others, such as Ref. [23], used more advanced RNN models. In Ref. [20], CNNs were used to extract features from the frames of a series of videos, which were then used as input to an LSTM model.

On the other hand, Ref. [56] presented a method for dynamic sign classification that involves extracting a sequence of frames, followed by a segmentation process using neural networks based on color, resulting in the segmentation of the skin of the hands and face. To classify the signs, four classical machine learning algorithms are compared, i.e., Bayesian classifier, decision trees, SVM, and NN.

Although research on LSM recognition has been conducted for several years, progress in this area has been slow and limited compared to other SLs. A common approach is to use computer vision techniques such as CNNs to build automatic sign recognition systems. However, with the recent emergence of pose recognition models, such as MediaPipe and YOLOv8, there is a trend in both LSM and other sign languages to use these tools to train more complex models, such as RNNs, or more sophisticated architectures, such as Transformers. A comparison of the studies mentioned here, with additional details, is shown in Table 4.

**Table 4:** LSM research.

Ref.	Sign Group *	Sign Type	Sign Feature	Sensor/Tool
Solís et al. (2016) [42]	L	Static	HC	Camera
Carmona-Arroyo et al. (2021) [43]	L	Static	HC	Leap Motion, Kinect
Salinas-Medina and Neme-Castillo (2021) [44]	L	Static	HC	Camera
Rios-Figueroa et al. (2022) [45]	L	Static	HC	Kinect
Morfín-Chávez et al. (2023) [46]	L	Static	HC	Camera/MediaPipe
Sánchez-Vicinaiz et al. (2024) [47]	L	Static	HC	Camera/MediaPipe
García-Gil et al. (2024) [48]	L	Static	HC	Camera/MediaPipe
Jimenez et al. (2017) [49]	L, N	Static	HC	Kinect
Martínez-Gutiérrez et al. (2019) [51]	L	Both	HC	RealSense f200
Rodriguez et al. (2023) [52]	L, N	Both	HC	Camera/MediaPipe
Rodriguez et al. (2025) [57]	L, N	Both	HC	Camera/MediaPipe
Martinez-Seis et al. (2019) [50]	L	Both	AM	Camera
Mejía-Peréz et al. (2022) [23]	L, W	Both	HC, AM, NHG	OAK-D/MediaPipe
Sosa-Jiménez et al. (2022) [58]	L, N, W	Both	HC, body but not NHG	Kinect
Sosa-Jiménez et al. (2017) [53]	W, P	Dynamic	HC, AM	Kinect/Pose extraction
Varela-Santos et al. (2021) [59]	W	Dynamic	HC	Gloves
Espejel-Cabrera et al. (2021) [56]	W, P	Dynamic	HC	Camera
García-Bautista et al. (2017) [54]	W	Dynamic	AM	Kinect
Martínez-Guevara and Curiel (2024) [60]	W, P	Dynamic	AM	Camera/OpenPose
Martínez-Guevara et al. (2019) [61]	W	Dynamic	HC, AM	Camera
Trujillo-Romero and García-Bautista (2023) [55]	W, P	Dynamic	HC, AM	Kinect
Martínez-Guevara et al. (2023) [62]	W, P	Dynamic	HC, AM	Camera
Martínez-Sánchez et al. (2023) [20]	W	Dynamic	HC, AM, NHG	Camera
González-Rodríguez et al. (2024) [63]	P	Dynamic	HC, AM, NHG	Camera/MediaPipe
Miah et al. (2024) [35]	L, W, P	Dynamic	HC, AM, NHG	Camera/MediaPipe, OpenPose
Current study	W, P	Dynamic	AM	Camera/YOLOv8

* L: alphabet letter; N: number; W: word; P: phrase.

### 1.1. Toward a Recognition System for LSM

We present the sign data acquisition, the hardware selected, and the fundamental concepts of our research toward a recognition system for LSM.

#### 1.1.1. Contactless Sign Data Acquisition with Simple Hardware

Due to the socioeconomic conditions of the main users of LSM, this research uses contactless, simple hardware for sign data acquisition (i.e., a pure vision-based approach), since color cameras are widely accessible and available in portable devices, which are very common in Mexico. As presented in Table 4, one important remark is that only one LSM research work [59] used contact sensing for sign data acquisition.

#### 1.1.2. Sign Features

From a linguistics perspective, LSM signs present six documented parameters, that is, basic articulatory parameters that simultaneously combine to form signs [39,64,65,66]. We propose a simplified Kinematics perspective, as shown in Figure 1, which combines four of those parameters into arm movements (AMs):

1. Hand configuration (HC): The shape adopted by one or both hands. As seen in Table 1 and Table 3, most research focuses on HC only. Hand segmentation [67] and hand pose detectors are very promising technologies for this feature. The number of HCs required to perform a sign is variable in LSM; some examples regarding the number of HCs required for a sign are as follows: number “1” (1 HC), number “9” (2 HCs), number “15” (2 hands, 1 HC), and “grandmother” (2 hands, 3 HCs). See Appendix A for samples of these signs.
2. Non-hand gestures (NHGs): Facial expressions (frowning, raising eyebrows), gestures (puffing out cheeks, blowing), and body movements (pitching, nodding). While most signs do not require non-hand gestures, some LSM signs do. Some signs that require one or more NHGs are as follows: “How are you?”, “I’m sorry”, “Surprise!” (two NHGs of this sign are shown in Figure 1). See Appendix A for links to samples of these signs.
3. Arm movement (AM): This can be characterized by tracking the joint movements of wrists, shoulders, and elbows. It is enough to obtain the following basic articulatory parameters [39,64,65,66]:
  (a) Articulation location: This is the location on the signer’s body or space where the signs are executed.
  (b) Hand movement: The type of movement made by the joints from one point to another.
  (c) Direction of movement: The trajectory followed by the hand when making the sign.
  (d) Hand orientation: Orientation of the palm of one or both hands, with respect to the signer’s body when making the manual configuration.
  This part can be studied from pose-based approaches (c.f. [31,32] with pose estimation using AlphaPose).

Other decompositions have been proposed to simplify sign analysis, such as in [62] (Figure 1), where an LSM sign is decomposed into fixed postures and movements. We consider the fact that this approach could lose important information, as transitions in hand postures are also important (as documented in the Hamburg Notation System (HamNoSys)) [68].

The use of pose estimators, particularly MediaPipe, enables the extraction of facial, hand, and body features; c.f. [17,23]. The use of pose estimators is quite frequent in SL research, however, there are still areas for improvement (c.f. [27] (Figure 8)), where a PhBFC was designed to improve MediaPipe hand pose estimation. Complementary approaches like bimodal frameworks [17] highlight the current limitations of these estimators.

We believe that focusing on a single element to describe LSM is inadequate, given its meaning and contribution to the sign. But covering everything at the same time is also very complex, as seen in most LSM research. Since most of the LSM work focuses on HC, this paper focuses on the AM part and reports the approach created to analyze visual patterns in arm joint movements. Our current work uses YOLOv8 [69,70] for pose estimation. While it is a 2D method, and MediaPipe is better for 3D, we discuss our decision in Appendix B.

The main contribution of this work involves the use of arm movement keypoints, particularly wrist positions, as a partial feature for sign language recognition. This is motivated by the observation in [23], where wrist location played a crucial role in distinguishing similar signs. For instance, the same hand configuration used at different vertical positions (e.g., near the head to indicate a headache, or near the stomach to indicate a stomachache) conveys different meanings. By isolating and analyzing this spatial feature, we aim to better understand its discriminative power in sign recognition tasks.

This paper is structured as follows. Section 2 describes the data acquisition, the experimental design and setup, the stages of the proposed approach for SLR, and the evaluation process and metrics. Section 3 describes the results from the analysis of two case studies and presents a comparison of the proposed methodology against state-of-the-art works. The potential and the limitations of our approach are discussed in Section 4. The conclusions of this work are presented in Section 5.

## 2. Materials and Methods

This section describes the resources, tools, and procedures used in this study. First, the acquisition of a visual sign language dataset is presented, including a detailed description of its features. Next, the experimental design is introduced, indicating the experiments to be performed and their objectives. Then, the experimental setup involving the deep learning models and the computational resources employed is described. Afterward, the stages of our first-step sign language recognition system are explained. Lastly, a detailed explanation of motion shapes used in the experimentation and the evaluation metrics is provided.

### 2.1. Data Acquisition

In this research, a proprietary dataset was developed with the active participation of the deaf community and LSM experts, ensuring no restrictions on recognizing hand configurations, arm movements, and facial expressions. The creation of the dataset was reviewed and approved by the Bioethics Committee for Human Research at Cinvestav, and all participants provided written informed consent.

The dataset comprises 74 signs—73 performed by 17 subjects and 1 (“iron”) performed by 16 subjects. In total, we have 1257 color videos (900 × 720 @ 90 fps) for RGB data acquisition. We consider this dataset a visual sign signal dataset.

All signs show HCs and AM, and three of them have NHGs (“How?”, “How are you?”, “Why?”). There are four phrases in the dataset, as follows: “Good morning!” (“*¡Buenos días!*”), “Good afternoon!” (“*¡Buenas tardes!*”), “How are you?” (“*¿Cómo estás?*”), and “Why?” (“*¿Por qué?*”). The latter is a question word in English, but it is constructed with two words in Spanish and, in LSM, is represented by a sign composed of two signs with independent meanings. This information is summarized in Table 5.

### 2.2. Experimental Design

Experiments were conducted on the custom dataset. The goal of these experiments was to classify dynamic LSM signs by detecting and tracking the wrist, elbow, and shoulder joints in order to characterize the AM. For this purpose, since sign production involves motion and changes in shape in space, we decided to use a pose-based approach to transform the visual sign signals into keypoint sign signals, and CNN for classification.

Two case studies are presented in this research. The first case only considers shoulders and wrists, as the wrists exhibit the predominant movement while the shoulders serve as base joints with minimal displacement. The second case includes the elbows, in addition to the shoulders and wrists, as the elbows also experience significant movement.

To carry out these analyses, three groups of signs were selected from the custom dataset. Each group was chosen based on specific characteristics. The first two subsets were selected based on signs with visually distinguishable motion patterns; in contrast, the third subset is composed of signs with variants to examine how this variability influences the classifier’s performance. More detailed information about these subsets is provided in Section 2.5.

### 2.3. Experimental Setup

For the experimentation, a pose detector and a CNN classifier framework were required. To select a pose estimation framework, we conducted preliminary experiments to compare the commonly used MediaPipe (Google LLC, Mountain View, CA, USA) and the YOLOv8-pose (Ultralytics Inc., Frederick, MD, USA) detector. Based on this comparison, we chose YOLOv8-pose due to its superior performance. The details of this comparison, which support our decision, can be found in Appendix B.

As YOLOv8-pose was selected for pose estimation, we used YOLOv8-cls (Ultralytics Inc., Frederick, MD, USA) to analyze visual patterns of the arm joint movements. Using a single technology for multiple tasks offers several advantages. For example, a unified architecture reduces the need for format adaptation between different models, simplifies implementation, and streamlines the workflow. Also, it reduces the possible problems of training and running multiple models across different frameworks.

A micromamba (QuantStack, Saint-Maur-des-Fossés, France) environment was employed for the installation and implementation of the pose detection and image classification models used in this work. Table 6 provides a summary of the technical specifications of the components of the experimental setting.

### 2.4. Sign Language Recognition

**Figure 2:**
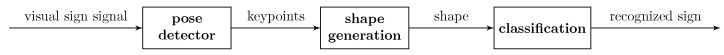
Pipeline of the arm movement approach for SLR.

This work represents a preliminary step toward a recognition system, which comprises a three-step process, as follows: (1) pose estimation, (2) shape generation, and (3) class prediction. A simplified diagram of this process is presented in Figure 2.

In this setup, a video file is passed through a pose detector, where six keypoints are extracted for each frame and saved as a NumPy (NumFOCUS, Austin, TX, USA) array. These keypoints are then plotted to generate motion shapes, and the resulting images are used as input to a classification model. The classification model returns the top five predicted classes and their associated confidence score. Detailed descriptions of each stage in the process are provided in the following subsections.

#### 2.4.1. Visual Sign Signals

**Figure 3:**
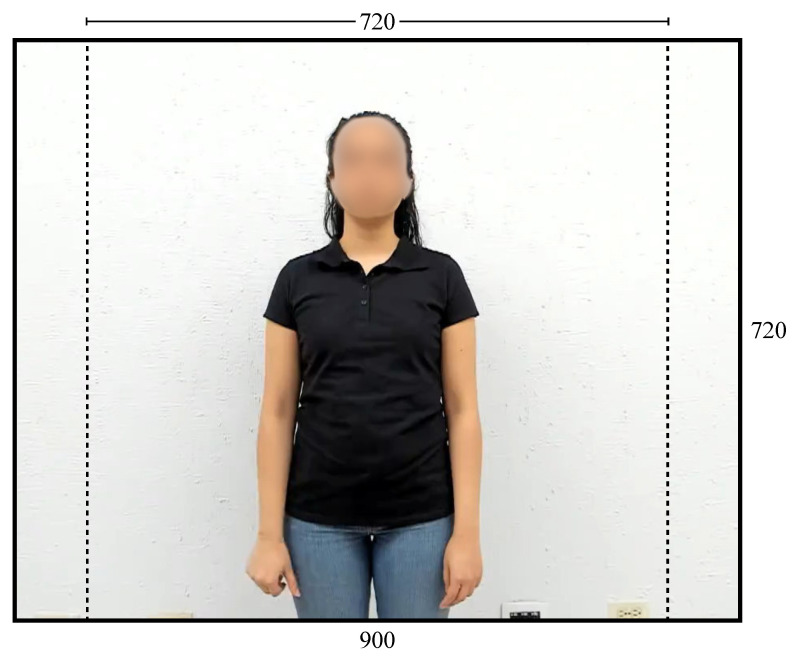
Dimensions of original and cropped frames.

To process the visual information, the video frames were cropped to 720 × 720 pixels (see Figure 3), as YOLOv8-pose operates internally on square images. This adjustment does not affect sign visibility, as all relevant joints remain within the square frame.

#### 2.4.2. Pose Estimation

**Figure 4:**
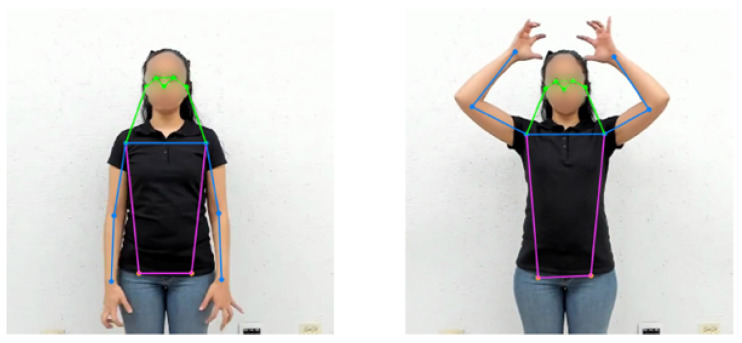
Pose detection of the “deer” sign. (**Left**): neutral pose. (**Right**): final pose.

LSM specifies that only the upper part of the body is meaningful in signing; so from the 17 keypoints detected by the selected pose detector, only 13 corresponding to the upper body are relevant; the 4 keypoints for knees and ankles are discarded. If the model fails to detect a joint, it is assigned a null value, which allows for easily discarding these missing values in further processing. Below is an example of pose estimation applied to the initial and final poses of the “deer” sign (Figure 4), as well as the extraction of the 13 keypoints.

The keypoints are stored in NPY format, a file type used by NumPy for efficiently storing data arrays. These arrays have dimensions of (13, 2, N): keypoints, 2D (x,y) coordinates, and the number of frames in each video.

#### 2.4.3. Shape Generation

**Figure 5:**
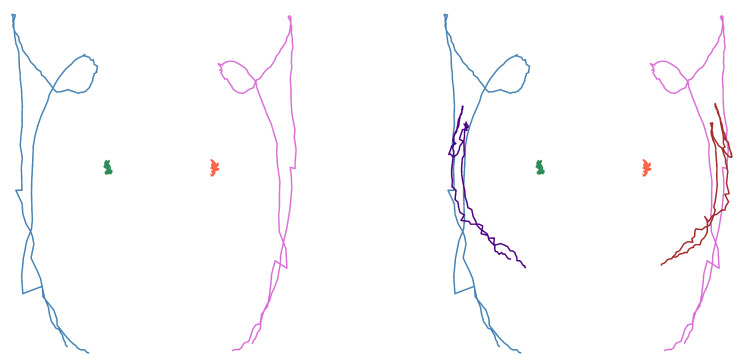
Movement shapes for the “deer” sign. (**Left**): only wrists and shoulder. (**Right**): also elbows.

From these arrays, the coordinates corresponding to the wrists, shoulders, and elbows are extracted according to each case study. The positions of these coordinates were plotted for each frame, illustrating the movement pattern of each joint, as shown in Figure 5.

#### 2.4.4. Classification

The shape classification stage involves assigning each image a label from a predefined set of classes. For this purpose, the YOLOv8x-cls model was employed. This classifier is the most robust of the YOLOv8 classification models and maintains a deep CNN structure. The classifier outputs the top-5 predicted class labels along with their associated confidence scores.

The maximum number of examples per sign in all selected sets is 17; 10 examples were used for training, 2 for validation, and 5 for the testing phase. Table 7 shows the most relevant hyperparameters for model training and configuration. On the other hand, Table 8 details the data augmentation-related hyperparameters handled by YOLOv8 (not all parameters are active).

### 2.5. Evaluation

Experimentation was conducted on the two case studies outlined in Section 2.2, using three sets of motion shapes described below. The lists of signs in each subset are shown in Table 9, Table 10 and Table 11

**Figure 6:**
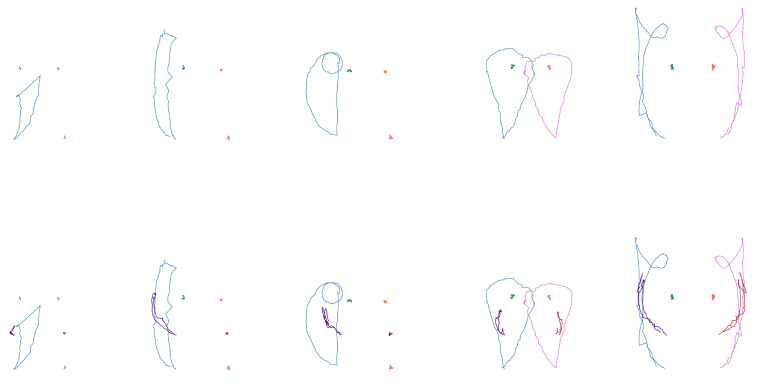
Shapes of the first subset (see words in Table 9). (**Top**): only wrists and shoulders. (**Bottom**): also elbows.

The first subset consists of a small group of five signs, chosen for their distinguishable shapes based on a qualitative evaluation. The primary objective of this group is to conduct a more controlled evaluation of the neural network, which allows for a clearer analysis of what the network is learning in an environment with fewer variables. Examples of these signs are presented in Figure 6, while the corresponding words are listed in Table 9.

**Figure 7:**
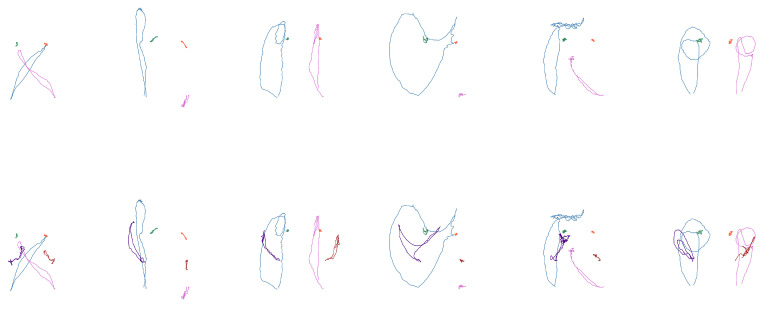
Shape examples of the second subset (“hug”, “tall”, “atole”, “airplane”, “flag”, and “bicycle”). (**Top**): only wrists and shoulders. (**Bottom**): also elbows.

In the second subset, the signs are similarly distinguishable, but with a larger set consisting of 62 signs. The goal now is to assess whether the neural network’s behavior remains consistent with that of the first set, despite the increased number of classes. Some examples of these signs are presented in Figure 7, and the corresponding words are listed in Table 10.

**Figure 8:**
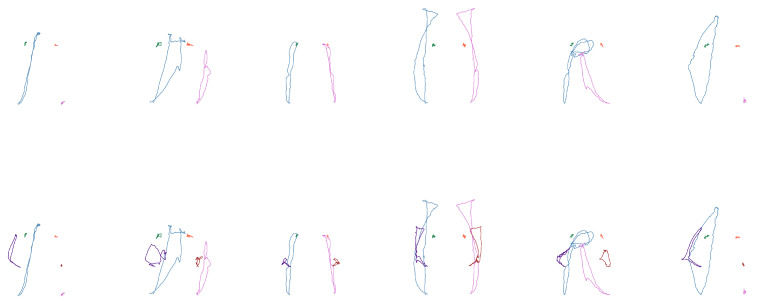
Shape examples of the third subset (“garbage”, “trash can”, “house”, “curtains”, “electricity”, and “stairs”). (**Top**): only wrists and shoulders. (**Bottom**): also elbows.

The third subset consists of 16 words related to the semantic field of house. This group is particularly notable for the high number of variants in its signs. As such, this experiment aims to assess the model’s accuracy, as well as its ability to generalize and identify distinctive features within more complex sign language contexts. Examples of the sign forms from this set can be seen in Figure 8, and the corresponding vocabulary is outlined in Table 11.

Once the training stage is completed, the corresponding weights are saved in a custom model, which is then utilized for the subsequent testing phase. During this phase, key performance metrics, such as top-1 and top-5 accuracies, are collected. Top-1 accuracy measures how often the model’s first prediction is correct, while top-5 accuracy evaluates whether the correct class appears among the five most probable predictions.

Top-1 accuracy is computed using the standard approach employed for most classification tasks. It is defined as the proportion of correctly predicted labels over the total number of samples. Let ${\hat{y}}_{i}$ be the predicted label for the *i*-th sample and $y_{i}$ the corresponding true label. The top-1 accuracy is then calculated as follows [71]:

(1) $${\text{top-1}}_{\mathrm{accuracy}}\left(y,\hat{y}\right)=\frac{1}{n_{\mathrm{samples}}}\sum_{i=0}^{n_{\mathrm{samples}}-1}1\left({\hat{y}}_{i}=y_{i}\right)$$

where 1(x) is the indicator function, which returns 1 if the prediction is correct and 0 otherwise.

On the other hand, the top-k accuracy considers a prediction correct if the true label is among the *k*-highest predicted scores. Thus, top-1 accuracy is a special case of top-k accuracy, where k=1.

Let ${\hat{f}}_{i,j}$ represent the predicted class for the *i*-th sample that has the *j*-th highest predicted score, and let $y_{i}$ be the corresponding true label. The top-k accuracy is then calculated as follows [72]:

(2) $${\text{top-k}}_{\mathrm{accuracy}}\left(y,\hat{f}\right)=\frac{1}{n_{\mathrm{samples}}}\sum_{i=0}^{n_{\mathrm{samples}}-1}\sum_{j=1}^{k}1\left({\hat{f}}_{i,j}=y_{i}\right)$$

where *k* is the number of top predictions considered, and 1(x) is the indicator function.

These metrics are crucial for assessing the model’s performance in a multi-class classification environment.

Additionally, a confusion matrix is generated for each experiment, providing a detailed overview of correct and incorrect predictions for each class. The results, along with their interpretation and analysis, are discussed in the following section.

## 3. Results

A total of seven SLR experiments on LSM were conducted (six with our custom dataset and one using an external dataset) to test our approach. The results are presented below.

Performance was evaluated using top-1 accuracy, top-5 accuracy, and the confusion matrix (see Section 2.5), which together provide a comprehensive view of the model’s performance across each subset. In addition, performance graphs depicting loss and accuracy across training epochs are included, allowing observation of the model’s learning curve over time.

### 3.1. Visual Sign Signal Dataset

#### 3.1.1. First Subset

**Figure 9:**
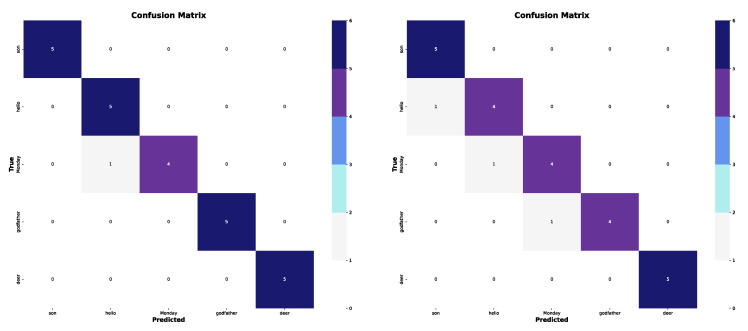
Confusion matrices for the first subset. (**Left**): only wrists and shoulders. (**Right**): also elbows.

In the first experiment, five of the most distinguishable classes were selected (see confusion matrices in Figure 9). The results reveal that using only the shoulder and wrist coordinates achieved a top-1 accuracy of 0.9599. However, when the elbow coordinates were included, the top-1 accuracy decreased to 0.8799, suggesting that the additional information had a negative impact on performance.

**Figure 10:**
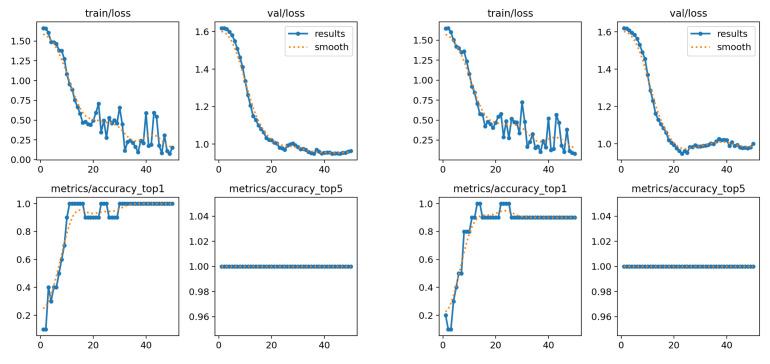
Performance charts for the first subset. (**Left**): only wrists and shoulders. (**Right**): also elbows.

Both the “son” and “deer” classes were classified with high accuracy in both case studies. However, slight confusion was observed between the “Monday” and “hello” classes in the first case. Additionally, when elbow coordinates were included, the model made errors in three of the five classes, indicating greater difficulty in differentiating between them. The performance graphs show that the accuracy in both models tends to stabilize around the 30th epoch, while the loss continues to decrease. Despite this, the model using only the wrist and shoulder coordinates outperformed the version with elbow coordinates, achieving higher accuracy (see graphs in Figure 10). In summary, the results are highly favorable in the best-case scenario, with a classification rate exceeding 95%. This suggests that the model is capable of effectively distinguishing between a limited number of well-defined classes. However, it is preferable to restrict the analysis to wrist and shoulder data, as including elbow data appears to negatively impact performance.

#### 3.1.2. Second Subset

**Figure 11:**
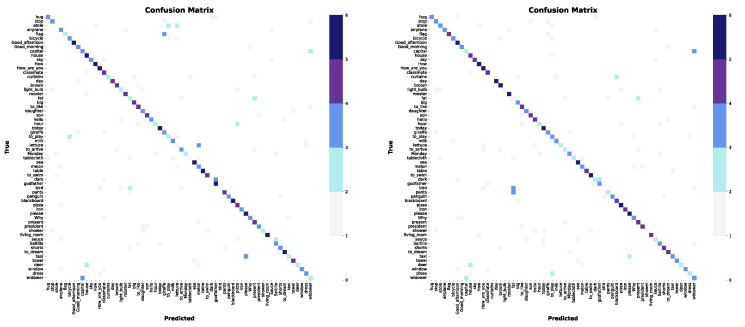
Confusion matrices for the second subset. (**Left**): only wrists and shoulders. (**Right**): also elbows.

In the second experiment, we expanded the number of classes to 62, while ensuring that the shapes remained distinguishable from one another (see confusion matrices in Figure 11). The model using only wrist and shoulder coordinates achieved a top-1 accuracy of 0.6375, whereas including elbow information resulted in a slight improvement to 0.6537.

**Figure 12:**
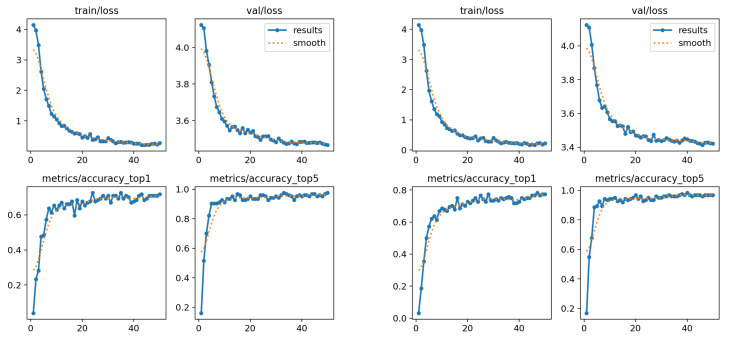
Performance charts for the second subset. (**Left**): only wrists and shoulders. (**Right**): also elbows.

For top-5 accuracy, the results were similar, with the first model achieving an accuracy of 0.8640, which improved to 0.8932 when elbow data was included. Performance analysis during training and validation revealed a consistent trend in both models, that is, accuracy steadily increased while loss progressively decreased (see Figure 12), indicating effective learning. The best model achieved an overall accuracy of 65%, which is acceptable, but showed variability in class performance. Some classes were classified nearly perfectly, while others exhibited notable precision issues. This suggests that, despite clear visual distinctions between classes, the large number of classes (62) combined with the limited number of examples per class (5) may hinder the model’s ability to generalize effectively. In conclusion, although incorporating elbow information improves classification accuracy, the inconsistent performance underscores the need for more examples per class to optimize the model’s results.

#### 3.1.3. Third Subset

**Figure 13:**
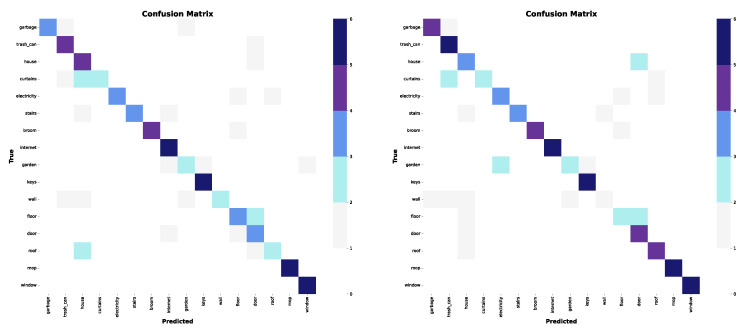
Confusion matrices for the third subset. (**Left**): only wrists and shoulders. (**Right**): also elbows.

In this experiment, the set is composed of 16 words in the home semantic field. The complexity of this group lies in the fact that some signs have variants. It is interesting to note that—in both models—words such as “internet”, “keys”, “mop”, and “window” were classified correctly since they showed less variability. In contrast, words like “curtains”, “garden”, and “wall” performed poorly, with poor predictions in both models (see confusion matrices in Figure 13).

The model using only wrist and shoulder information achieved a top-1 accuracy of 0.6875, while including the elbow coordinates increased the accuracy to 0.7125. For top-5 accuracy, both models achieved a value of 0.9250.

**Figure 14:**
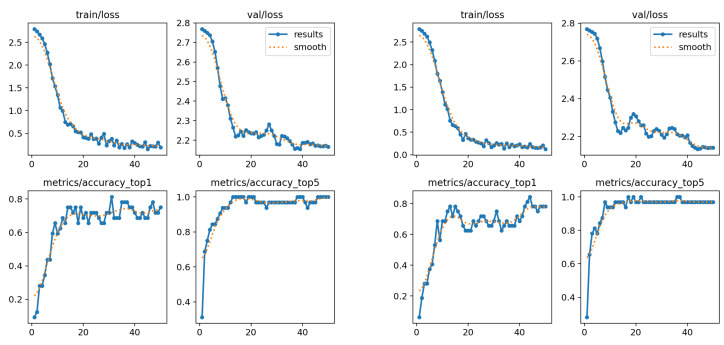
Performance charts for the third subset. (**Left**): only wrists and shoulders. (**Right**): also elbows.

Performance in both studies was quite similar (see the graphs in Figure 14), showing fluctuations during training, but with a tendency to stabilize at a constant value toward the later stages. This suggests that the model managed to learn the main features of the characters, although its generalization capacity is limited by the complexity of the variants within the set. The classification rate reached up to 71% when the elbow information was included, which indicates that this additional information contributes positively to the recognition, although the increase in accuracy is not very significant.

Despite the limitations, the model was able to detect patterns in some cases. However, its ability to generalize across a large number of classes, variants, and a limited number of examples is insufficient. Notwithstanding, the performance graphs reveal a tendency toward stabilization, suggesting that while the model holds potential for certain datasets, it requires additional information—such as finger movements—to enhance its classification accuracy in more complex scenarios.

### 3.2. Comparison of the Proposed Model on a Keypoint Sign Signal Dataset

**Figure 15:**
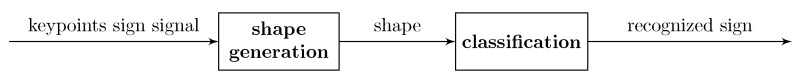
Pipeline of the arm movement approach for SLR with the MSLR dataset.

In order to compare our approach against other state-of-the-art works, we needed to perform additional experiments on another LSM dataset. We selected from among the publicly available LSM datasets; see Table 2 and Table 3. Our selection criterion was based on the number of SLR studies that used each dataset and reported performance accuracy, to enable a proper comparison. Therefore, we opted for a keypoint sign signal dataset, that is, the MSLR dataset from [23]. Details about this dataset are available in Appendix C. This dataset has been tested and reported by at least three different machine learning models [23,35,73]. In contrast to the visual sign signal dataset, the MSLR dataset required a shorter pipeline compared to the one required for visual signals. The pipeline is shown in Figure 15.

For this comparison, we tested our arm movement approach with this dataset using all the arm joints. For classification, we trained a model from scratch, using the current YOLO nanoarchitecture [74] YOLO11n-cls, with the PyTorch framework. This architecture uses 86 layers and has a computational complexity of 0.5 GFLOPs, with 1.633584 million parameters, when using a frame size of 224 pixels [75]. The results of this comparison are presented in Table 12.

## 4. Discussion

Table 13 presents the accuracy values based on the top-1 accuracy metric obtained using the YOLOv8x-cls model. The results indicate that including elbow coordinates led to better performance in two out of the three experiments. Although the improvement was modest (ranging from 3% to 4%), it suggests that incorporating additional joint information can contribute to more accurate classifications.

The experiments with various datasets allowed us to observe the behavior of the convolutional neural network (CNN) based on the input data. It became evident that the network’s performance is heavily influenced by the selection of classes. Using all available classes from the database is not always ideal, as this tends to yield suboptimal results. Therefore, a more focused approach, where only relevant classes are included, is recommended for improving model classification.

Despite certain limitations—such as the small number of examples per class, the presence of variants, and the high similarity between some signs—the neural network was still able to classify a significant number of signs correctly and recognize patterns in the movement data. This demonstrates the potential of the YOLOv8 model for this type of task.

Compared to other CNNs, YOLOv8 stands out due to its optimized architecture, which allows for the use of pre-trained models on large datasets like ImageNet. This enables the model to achieve high accuracy and efficiency, making it suitable for real-time applications. However, as with any model, performance is largely dependent on the quality and quantity of the input data. In this case, the limited number of examples (17 per class) restricts the network’s ability to achieve optimal accuracy.

These results highlight both the potential and the limitations of our approach. The experiments demonstrated that it is possible to classify a considerable number of signs, indicating that this dataset and strategy could serve as a useful tool for training a convolutional neural network (CNN), such as YOLOv8. However, the analysis also reveals that the current structure of the dataset—characterized by a limited number of examples, variants between classes, and high similarity among some signs—presents challenges that must be addressed through alternative approaches.

The comparison between the two case studies (with and without elbows) was intended to assess whether the inclusion of a greater number of keypoints improves the performance of the model. This seems to indicate that this assumption is correct. The next immediate step is to optimize these results, either by using a different convolutional neural network (CNN) or by exploring different architectures, such as recurrent neural networks (RNNs), but keeping the focus on the use of keypoints; i.e., using pose-based approaches.

Additionally, the study performed on the MSLR dataset showed good results for the proposed approach (with an accuracy of 85.78 % using 6 keypoints), compared to the extraordinary results obtained by [23] (with accuracies of 96.44 % and 97.11 %) and [35] (with accuracies of 99 % and 99.75 %) while using the complete keypoint sign signals in the dataset; see Table 12. This is an interesting finding that shows the relevance of AM sign features, as most previous research studies typically focused on HC sign features.

## 5. Conclusions

This paper presents ongoing work toward the creation of a recognition system for LSM. A decomposition of sign features is proposed into HC, AM, and NHG. Contactless, simple hardware was used for sign signal acquisition. A custom proprietary dataset of 74 signs (70 words and 4 phrases) was constructed for this research. In contrast to most LSM research, this paper reports an analysis focused on the AM part of signs, rather than on HC-focused or holistic approaches (HC + AM + NHG).

The analysis was conducted through a series of classification experiments using YOLOv8, aimed at identifying visual patterns in the movement of key joints, i.e., wrists, shoulders, and elbows. A pose detection model was used to extract joint movements, followed by an image classification model (both integrated into YOLOv8) to classify the shapes generated by these movements.

These experiments are the first stage of a larger project. For now, we are focusing on the analysis of arm movement (shoulders, elbows, and wrists) because it is a less-studied feature, and information can be extracted from it using a relatively simple methodology.

Later, the goal will be to integrate other essential components of sign language, such as manual configuration and non-hand gestures, to develop a more complete system. Ultimately, this will support progress toward automatic sign language recognition.

## Figures and Tables

**Table 5:** Custom dataset.

Feature	Description
Signs *	70 W, 4 P
Signers	17
Samples	73 signs with 17 samples, 1 sign with 16 samples
Sign features	HC, AM, NHG
Sign signal	Visual
Signal properties	900 × 720 @ 90 fps
File format	MKV videos
Samples for training	10 samples
Samples for validation	2 samples
Samples for testing	5 samples

* W: words; P: phrase.

**Table 6:** Computational resources.

Component	Version/Model
Operating system	Ubuntu 22.04.2 (Canonical Ltd., London, England)
Graphics card	Asus ROG STRIX GeForce RTX 2080 Ti O11G (ASUS Holdings Mexico S.A. de C.V., Mexico City, Mexico)
Computing API	CUDA 12.4 (NVIDIA Corporation, Santa Clara, CA, USA)
Programming language	Python 3.11.8 (Python Software Foundation, Beaverton, OR, USA)
Machine Learning library	PyTorch 2.2.2 (Linux Foundation, San Francisco, CA, USA)
Framework	YOLO 8.1.47 (Ultralytics Inc., Frederick, MD, USA)

**Table 7:** Training parameters and their descriptions.

Parameter	Value	Description
epochs	50	Number of epochs or training cycles.
batch	16	Number of images processed in each iteration.
imgsz	224	Size of the images input into the model.
patience	100	Number of epochs without improvement before stopping the training.
lr0	0.01	Initial learning rate.
pre-trained	True	Indicates that the model uses pre-trained weights (ImageNet).
single_cls	False	If set to true, the model classifies into a single class.
dropout	0.0	Dropout rate. This is a regularization technique used to reduce overfitting in artificial neural networks.

**Table 8:** Image augmentation parameters and their descriptions.

Parameter	Value	Description
hsv_h	0.015	Hue of the image in the HSV color space.
hsv_s	0.7	Saturation of the image in the HSV color space.
hsv_v	0.4	Brightness of the image in the HSV color space.
degrees	0.0	Random rotation applied to the images.
translate	0.1	Random translation of the images.
scale	0.5	Random scaling factor applied to the images.
shear	0.0	Random shear angle applied to the images.
perspective	0.0	Perspective transformation applied to the images.
flipud	0.0	Probability of flipping the image vertically.
fliplr	0.5	Probability of flipping the image horizontally.
bgr	0.0	BGR to RGB color space correction factor.
mosaic	1.0	Probability of using the mosaic technique to combine images.
mixup	0.0	Probability of mixing two images.
copy_paste	0.0	Technique of copying and pasting objects between images.
auto_augment	randaugment	Type of data augmentation used.
erasing	0.4	Probability of erasing parts of the image to simulate occlusions.
crop_fraction	1.0	Proportion of the image to be cropped. A value of 1.0 indicates no cropping.

**Table 9:** Signs for the first subset.

No.	Semantic Field	Sign
1	family	son *
2	greetings	hello *
3	days of the week	Monday *
4	family	godfather *
5	animals	deer *

* These signs are also in the second subset.

**Table 10:** Signs for the second subset.

No.	Semantic Field	Sign	No.	Semantic Field	Sign
1	verbs	hug	32	verbs	to arrive
2	adjectives	tall	33	days of the week	Monday *
3	drinks	atole	34	kitchen	tablecloth
4	transport	airplane	35	miscellaneous	sea
5	school	flag	36	fruits	melon
6	transport	bicycle	37	kitchen	table
7	greetings	Good afternoon!	38	verbs	to swim
8	greetings	Good morning!	39	colors	dark
9	cities	capital	40	family	godfather *
10	house ^†^	house	41	animals	bird
11	miscellaneous	sky	42	clothing	pants
12	questions	How?	43	animals	penguin
13	questions	How are you?	44	school	blackboard
14	school	classmate	45	food	pizza
15	house	curtains ^†^	46	room	iron
16	days of the week	day	47	miscellaneous	please
17	house	broom ^†^	48	questions	Why?
18	living room	light bulb	49	time	present
19	animals	rooster	50	professions	president
20	adjectives	fat	51	bathroom	shower
21	adjectives	big	52	living room	living room
22	verbs	to like	53	food	sauce
23	family	daughter	54	cities	Saltillo
24	family	son *	55	clothing	shorts
25	greetings	hello *	56	verbs	to dream
26	time	hour	57	transport	taxi
27	time	today	58	bathroom	towel
28	animals	giraffe	59	animals	deer *
29	verbs	to play	60	house	window ^†^
30	drinks	milk	61	clothing	dress
31	vegetables	lettuce	62	person	widower

* These signs are also in the first training set. ^†^ These signs are also in the third subset.

**Table 11:** Signs for the third subset.

No.	Semantic Field	Sign
1	house	garbage
2	house	trash can
3	house	house *
4	house	curtains *
5	house	electricity
6	house	stairs
7	house	broom *
8	house	internet
9	house	garden
10	house	keys
11	house	wall
12	house	floor
13	house	door
14	house	roof
15	house	mop
16	house	window *

* These signs are also in the second subset.

**Table 12:** Performance accuracy with the MSLR dataset and a state-of-the-art comparison.

Ref.	Dataset	Joint Keypoints *	Performance Accuracy (%)
RNN [23]	MSLR	67	96.44
GRU [23]	MSLR	67	97.11
Dynamic-GCN ^†^ [73]	MSLR	67	98.55
Single-stream GCAR [35]	MSLR	67	99.00
Two-stream GCAR [35]	MSLR	67	99.75
Proposed model	MSLR	6	85.78

* 67 keypoints of the full body; 6 keypoints of the arm joints: wrists, elbows, and shoulders. ^†^ The model is presented in [73]; the performance accuracy is reported in [35].

**Table 13:** Top-1 accuracy comparison on the custom dataset.

Dataset	No. Classes	Description	With Elbows	Without Elbows
1	5	More distinguishable	0.8799	0.9599
2	62	More or less distinguishable	0.6537	0.6375
3	16	House group	0.7125	0.6875

**Figure A1:**
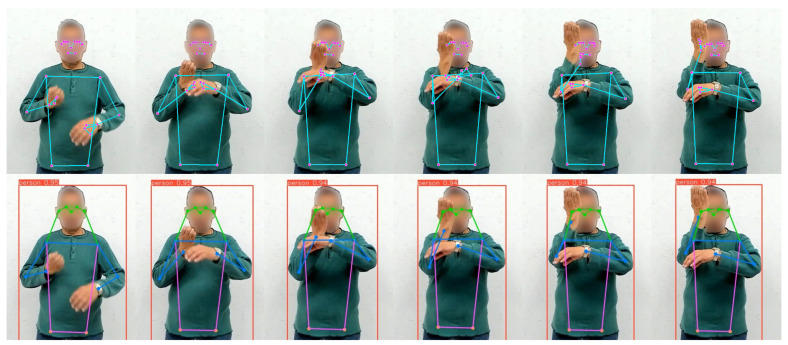
Comparison of wrist joint tracking between YOLOv8 and MediaPipe. Example with the “state” sign. Top row: MediaPipe. Bottom row: YOLOv8 pose detector. Four inner frames: MediaPipe loses track of the wrist joint, while YOLOv8 keeps track of the AM in all frames.

## Data Availability

The datasets presented in this article are not readily available because the data are part of an ongoing study, and due to technical and time limitations. Requests to access the datasets should be directed to keny.ordaz@cinvestav.edu.mx.

## Abbreviations

The following abbreviations are used in this manuscript:

AM	arm movement
API	application programming interface
ArSL	Arabic Sign Language
ASL	American Sign Language
BdSL	Bangladeshi Sign Language
CESAR	Recife Center for Advanced Studies and Systems
CSL	Chinese Sign Language
CSV	comma-separated values
CNN	convolutional neural network
DGS	German Sign Language (*Deutsche Gebärdensprache*)
EMG	electromyography
FLV	flash video
fps	frames per second, frame rate
GCAR	graph convolution with attention and residual connection
GCN	graph convolutional network
GRU	gated recurrent units

HC	hand configuration
IMU	inertial measurement unit
JPEG	Joint Photographic Experts Group, ISO/IEC 10918
LIBRAS	Brazilian Sign Language (*Língua Brasileira de Sinais*)
LSA	Argentinian Sign Language (*Lengua de Señas Argentina*)
LSE	Spanish Sign Language (*Lengua de Señas Española*)
LSM	Mexican Sign Language (*Lengua de Señas Mexicana*)
LSTM	long short-term memory
MKV	Matroska video
MLP	multilayer perceptron
MSLR	Mexican Sign Language Recognition dataset
MP4	MPEG-4 Part 14, ISO/IEC 14496-14:2003
NHG	non-hand gesture
NN	neural network
NPY	NumPy standard binary file format
PJM	Polish Sign Language (*Polski Język Migowy*)
PSL	Pakistan Sign Language
RGBD	red, green, blue, and depth
RNN	recurrent neural network
sEMG	surface EMG
SL	sign language
SLR	sign language recognition
SVM	support vector machine
SWF	small web format
TSL	Turkish Sign Language
YOLO	you only look once

## Appendix A. Digital Glossary of LSM

The GDLSM [38] has 747 signs grouped into 19 thematic categories. We provide direct links to some of the signs included in this digital glossary, which were mentioned in Section 1.1.2.

- Numbers (*Números*); https://lsm.indiscapacidad.cdmx.gob.mx/ejes/numeros/, accessed on 5 June 2025:
  – 1 (one): https://youtu.be/zcd4GfYz-fA, accessed on 5 June 2025.
  – 9 (nine): https://youtu.be/MgnvumQM-cQ, accessed on 5 June 2025.
  – 15 (fifteen) (first variant): https://youtu.be/yZ3X38cFWUM, accessed on 5 June 2025.
  – 15 (fifteen) (second variant): https://youtu.be/64jBCZXv6rY, accessed on 5 June 2025.
- Family (*Familia*); https://lsm.indiscapacidad.cdmx.gob.mx/ejes/familia/, accessed on 5 June 2025:
  – Grandmother (*abuela*): https://youtu.be/lckOvtr0lZU, accessed on 5 June 2025.
- Everyday expressions (*Expresiones cotidianas*); https://lsm.indiscapacidad.cdmx.gob.mx/ejes/expresiones-cotidianas/, accessed on 5 June 2025:
  – How are you? (*¿Cómo estás?*) https://youtu.be/x7zFMacTe04, accessed on 5 June 2025.
  – I’m sorry (*Disculpa*) https://youtu.be/bWwIisAtYCI, accessed on 5 June 2025.
  – Surprise! (*¡Sorpresa!*) https://youtu.be/Q0OqTBjoIjU, accessed on 5 June 2025, this sign was used in Figure 1.

## Appendix B. Comparison Between MediaPipe and YOLOv8 Pose Detection Models

MediaPipe detects 33 keypoints with its Pose Landmarker (Heavy) model, and it can provide 2D and 3D coordinates. YOLOv8 detects 17 keypoints with its YOLOv8x-pose-p6 model and provides 2D coordinates. YOLOv8 keypoints 5–10 are for the shoulder, elbow, and wrist joints, and MediaPipe keypoints 11–16 are for the same joints. We compared the MediaPipe and the YOLOv8 pose detectors in several signs. We decided to use YOLOv8 over MediaPipe due to frequent tracking failures of the wrist joint in many of the signs, particularly in the occluded conditions of the hands. An example of this issue is shown in Figure A1.

## Appendix C. MSLR Dataset

The Mexican Sign Language Recognition (MSLR) dataset was created by [23]. It contains samples of 30 signs in LSM; see Table A1. This is a keypoint-based sign signal dataset, as shown in Table A2. Each sample consists of 20 frames, with 67 pose keypoints recorded per frame. The keypoints are distributed as follows: 20 for the face, 5 for the body (shoulders, elbows, and a midpoint between the shoulders), and 21 for each hand.

**Table A1:** Signs in the MSLR [23] dataset.

No.	Group *	Sign
1	alphabet	A
2	alphabet	B
3	alphabet	C
4	alphabet	D
5	alphabet	J
6	alphabet	K
7	alphabet	Q
8	alphabet	X
9	questions	What?
10	questions	When?
11	questions	How much?
12	questions	Where?
13	questions	For what?
14	questions	Why?
15	questions	What is that?
16	questions	Who?
17	days of the week	Monday
18	days of the week	Tuesday
19	days of the week	Wednesday
20	days of the week	Thursday
21	days of the week	Friday
22	days of the week	Saturday
23	days of the week	Sunday
24	frequent words	(to) spell
25	frequent words	(to) explain
26	frequent words	thank you
27	frequent words	name
28	frequent words	please
29	frequent words	yes
30	frequent words	no

* Group names and information taken from [23] (Table 2).

**Table A2:** MSLR [23] dataset.

Feature	Description
Signs *	8 L, 21 W, 1 P
Signers	4
Samples	30 signs with 100 samples
Sign features	HC, AM, NHG
Sign signal	Keypoints
File format	CSV files
Samples for training ^†^	70 samples
Samples for validation ^†^	15 samples
Samples for testing ^†^	15 samples

* L: letters, W: words; P: phrase. ^†^ This split was defined by the dataset authors.
